# Multidrug Resistance Urinary Tract Infection in Chronic Kidney Disease Patients: An Observational Study

**DOI:** 10.7759/cureus.38571

**Published:** 2023-05-05

**Authors:** A.H.M Sanjedul Haque Sumon, Md. Rashid Al-Mahmood, Khaza Amirul Islam, A.N.M Ehsanul Karim, Parvin Aker, Ahsan Ullah, Mohammad Afzalur Rashid, Md Nazmul Hasan

**Affiliations:** 1 Nephrology, Shaheed Ziaur Rahman Medical College Hospital, Bogura, BGD; 2 Physical Medicine and Rehabilitation, Bangabandhu Sheikh Mujib Medical University, Dhaka, BGD; 3 Physical Medicine and Rehabilitation, Northern International Medical College, Dhaka, BGD; 4 Hematology, Shaheed Ziaur Rahman Medical College Hospital, Bogura, BGD; 5 Biochemistry, Shaheed Ziaur Rahman Medical College Hospital, Bogura, BGD; 6 Internal Medicine, Titas Upazila Health Complex, Cumilla, BGD; 7 Emergency Department, Directorate General of Health Services, Dhaka, BGD; 8 Internal Medicine, Bangabandhu Sheikh Mujib Medical University, Dhaka, BGD

**Keywords:** loin pain, antibiotic resistance, chronic kidney disease, urinary tract infection, multidrug resistance

## Abstract

Objective: To determine the presence of multidrug-resistant (MDR) urinary tract infections (UTI) and the MDR pattern of the bacterial isolates causing MDR UTI in chronic kidney disease (CKD) patients.

Methods: This cross-sectional study was conducted among 326 diagnosed CKD patients in the Department of Nephrology at Bangabandhu Sheikh Mujib Medical University (BSMMU). Purposive sampling technique was used, and data were collected from the respondents using a semi-structured questionnaire. From duly collected urine samples, identification of organisms and antibiotic susceptibility tests were done, maintaining proper procedure in the microbiology laboratory.

Results: The study population was predominantly female (60.1%). The outpatient department provided the majority of the respondents (75.2%). A history of UTI within the last six months was present among 74.2% of the respondents, and 59.2% had a history of taking antibiotics. Bacterial isolates were predominantly gram-negative (79.4%). *Escherichia coli* was the most prevalent bacterial isolate, present in 55.5% of the study population. Among the respondents, 64.7% were found to have MDR UTI, and among them, 81.5% were gram-negative, and 18.5% were gram-positive isolates. Among all the antibiotics tested, Colistin Sulphate, Polymyxin B, Cefoxitin, Vancomycin, and Linezolid had the highest (100%) sensitivity, followed by Meropenem, with 94.9% sensitivity. Among the gram-negative isolates, Acinetobacter and Enterobacter were most resistant to aminoglycoside, at 70% and 91.7%, respectively. *E. coli*, *Klebsiella*, *Proteus*, and *Pseudomonas* were most resistant to quinolone at 76.8%, 76.9%, 83.3%, and 66.7%, respectively. Among the gram-positive isolates, *Enterococci* and *Staphylococcus aureus* were most resistant to aminoglycoside, 81.5% and 88.9%, respectively. *Streptococcus* was found to be most resistant to cephalosporin (75.0%). There was a statistically significant (p < 0.05) relationship between MDR UTI, history of UTI, and previous antibiotic intake, and diabetic CKD.

Conclusions: The prevalence of MDR UTI among CKD patients is considerably high. When treating UTI, choosing an appropriate antibiotic by urine culture and implementing a guideline on the rational use of antibiotics are essential to managing and preventing the development of MDR UTI.

## Introduction

Urinary tract infection (UTI) is a term applied to a variety of clinical conditions ranging from the asymptomatic presence of bacteria in the urine to severe kidney infection with resultant sepsis [1]. Symptoms of a UTI could span a wide spectrum, ranging from mild irritative voiding to bacteremia, sepsis, shock, or even death. In particular patient groups, urosepsis may result in high mortality rates of 25% to 60% [2]. 

Risk factors like advanced age, diabetes, previous history of UTI, and an increasing number of invasive urologic procedures for both diagnosis and treatment have been related to the high rates of UTI [3]. UTI occurs following the movement of bacteria through the urethra to the bladder, occasionally with an ascending infection to the kidney [4].

Normal flora of the gut, vagina, and per urethral region are common bacteria found in the urine of the affected person. Women are more affected than men because of the shortness of the female urethra [5].

Patients suffering from diabetes mellitus (DM) have a higher chance of being affected by urinary tract infections (UTIs). Different deteriorations in the immune system, including humoral, cellular, and innate immunity, may contribute to the pathogenesis of UTI in diabetic patients. Higher glucose concentrations in urine may accelerate the growth of pathogenic bacteria. High renal parenchymal glucose levels create a favorable environment for the growth and multiplication of microorganisms. This might be one of the contributing factors to pyelonephritis and other renal complications, such as emphysematous pyelonephritis [6].

Antibiotic resistance has become one of the major healthcare problems worldwide [7]. These patients constitute a reservoir for the spread of multidrug-resistant (MDR) bacteria. UTIs have been associated with high rates of treatment failure, resulting in the development of antibiotic resistance and, subsequently, multidrug resistance [8]. Some bacteria are virulent and capable of having multidrug resistance to antibiotics. For example, *Escherichia coli* is a gram-negative bacteria that can generate a large spectrum of beta-lactam enzymes, making them resistant to most beta-lactam antibiotics [9].

Rates of antimicrobial resistance are directly proportional to the misuse of antibiotics [10]. The use of Fluoroquinolones and Penicillin, with any antimicrobial before UTI presentation, is strongly associated with developing resistance [11].

Chronic kidney disease is a clinical syndrome characterized by defects in kidney glomerular filtration resulting in reduced metabolic waste product clearance from the blood. Worldwide, about 6% to 40% of patients with CKD are susceptible to infection with UTI caused by extended-spectrum β-lactamase (ESBL)-producing gram-negative bacteria [12].

The uremic condition in CKD changes both cellular and humoral immunity, which increases the possibility of a broad range of infections. In addition to less host immunity, co-morbidities, especially diabetes, advanced age, and urinary tract obstruction, are common risks for UTI among CKD patients [13]. Due to urinary stagnation, alkalization of urine, and the absence of flushing action, the chance of urinary tract infection (UTI) in CKD among males is higher [14].

There have been studies regarding the prevalence of bacteria isolated from urinary tract infections and their antibiotic resistance patterns, but such studies are scarce among chronic kidney disease patients in Bangladesh.

This study aimed to determine the presence of MDR among the patients with UTI in CKD and the multidrug resistance pattern of bacterial isolates, which would be useful to select more targeted antibiotic regimens and avoid frequent treatment with irrational broad-spectrum antibiotics.

## Materials and methods

This cross-sectional study was conducted in the Department of Nephrology from September 2019 to August 2020 in a tertiary hospital in Bangladesh. CKD patients of both genders, ≥18 years, had ≥ 10 pus cells/per high power field (HPF). in unspun urine samples from patients attending outpatient departments (OPD) or admitted indoors were included in the study [14]. Patients suffering from polymicrobial infections, including >2 bacterial species, infections with Candida species, concurrent infections other than UTI, or who were renal allograft recipients or pregnant, were excluded. Patients were fully informed about the procedure using their own language. Then, written consent was obtained for the study.

Using the purposive sampling technique, 376 eligible samples were enrolled in the study according to the inclusion and exclusion criteria, but 50 of those respondents dropped out during the study period. So, the final sample size was 326.

A semi-structured questionnaire and checklist were used to collect the data.

Urine collection technique

A clean midstream urine sample was collected in the morning. Before collection, patients were instructed to wash their hands and then the site of the external urethral meatus and perianal region with soap and warm water and then dry. In the case of an indwelling catheter, disinfecting the catheter collection port with 70% alcohol was done. We clamped the catheter at 5cm distal to the port for 15 min. Then we collected 10-15 ml of urine using a syringe aseptically. Patients’ full names and IDs were labeled on the container. The urine sample was transferred within two hours to the microbiology laboratory for culture and sensitivity testing in the department of microbiology of the same institution.

Identification of organisms

From each urine sample, about 5µl of urine was inoculated in the chromogenic media by a 2mm-diameter platinum wire loop at a 45-degree angle. After 24 hours of incubation at 37°C, families of different bacteria were identified by colony morphology on chromogenic media.

The chromogen X-glucosidase is cleaved by the beta-glucosidase enzyme, which is produced by *Enterococci*, resulting in a blue colony. The chromogen red-galactosidase is cleaved by the beta-galactosidase enzyme, which is produced by *E. coli*, resulting in a burgundy or pink colony. Cleavage of both chromogens by the coliform group results in a purple colony. *Proteus* and *Pseudomonas* produce brown and fluorescent colonies. *Staphylococcus aureus* produces white/creamy colonies.

Antimicrobial susceptibility test

Disk diffusion method (according to Clinical Laboratory Standards Institute (CLSI) guideline disk diffusion breakpoint criteria) was used for antimicrobial susceptibility testing of the isolated organism by the “Kirby-Bauer method” using Mueller-Hinton agar and commercially available antibiotic disc [15]. The dried surface of the Mueller-Hinton agar plate was inoculated by streaking the swab over the entire sterile agar surface. Predetermined antimicrobial disks were dispensed on the surface of the inoculated agar plate. Antibiotic discs of Ceftriaxone (30 𝜇g), Ceftazidime (30 μg), Cefotaxime (30 𝜇g), Cefepime (30 𝜇g), Cotrimoxazole (1.25/23.75 𝜇g), Ciprofloxacin (5 𝜇g), Gentamycin (10 𝜇g), Amikacin (30 𝜇g), Netilmicin (30 𝜇g), Imipenem (10𝜇g), Meropenem (10𝜇g), Piperacillin-Tazobactam(100/10𝜇g), Ticarcillin (100 µg), Colistin (10 𝜇g), Polymyxin B (10 µg), Tigecycline (15𝜇g) were used. The plates were inverted and placed in an incubator set to 37°C for another 16-18 hours. The disc content and zone of inhibition were used as recommended by the CLSI. In the case of Colistin and Polymyxin B, the zone of inhibition was used as per another study because such a guideline is absent in the CLSI guideline [16]. The antimicrobial discs were used according to the standard antibiotic panel for specific samples and isolated organisms. The zone of inhibition was fixed based on bacteria isolated in the urinary sample.

Data collection and analysis

Data were collected through face-to-face interviews, clinical examinations, and laboratory reports. The privacy of the data was strictly maintained. An ID number was given to the participant. 

The collected data were checked, verified, and then entered into the computer. The analysis was carried out with the help of IBM Corp. Released 2017. IBM SPSS Statistics for Windows, Version 25.0. Armonk, NY: IBM Corp. and Microsoft Excel (Redmond, USA) Version 2019 for Windows 10. The analysis was done according to the objectives. For descriptive statistics, frequency, percentage, and mean were used. Inferential statistics were carried out to see if there was any association between independent and dependent variables. For the test of significance, the Chi-square test was done to see the association between qualitative variables. A p-value of <0.05 was considered statistically significant.

Ethical implication

Ethical clearance was obtained from the Institutional Review Board (IRB) of Bangabandhu Sheikh Mujib Medical University (BSMMMU) (ID-BSMMU/2020/52, date of approval: 2-1-2020). There was no physical, psychological, or social risk to the patients. Informed and understood written consent was taken from every patient before enrollment. Privacy, anonymity, and confidentiality of data information identifying any patient were maintained strictly. Each patient enjoyed every right to participate, refuse, or even withdraw from the study at any point in time. The study conforms to the code of ethics of the World Medical Association (Helsinki Declaration).

## Results

Results are expressed in tables and figures. Table 1 shows that 60.1% of subjects were female. Most of the subjects (75.2%) were OPD-based. Fever, dysuria, and loin pain were more experienced clinical features. A history of UTI was present among 74.2% of the respondents.

**Table 1:** Descriptive statistics of study population (N = 326)

Criteria		Total	
		n	%
Sex	Male	130	39.9
	Female	196	60.1
	Total	326	100.0
Admission Status of the Patient	In-Patient	81	24.8
	Out-Patient	245	75.2
Clinical Features	Fever	231	70.9
	Dysuria	210	64.4
	Loin Pain	90	27.6
	Hematuria	11.0	3.4
	Nausea Vomiting	204	62.6
Past History (up to 6 months)	History of Diagnosed UTI	242	74.2
	History of Taking Antibiotic	193	59.2

**Figure 1:**
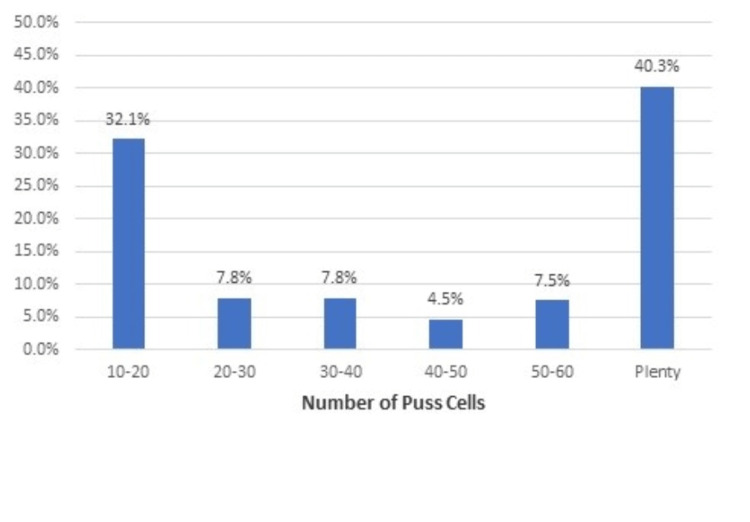
Distribution of the study population according to the number of pus cells per high-powered field (HPF) in the urine sample

Figure 1 showed that among the study population, the highest proportion, 40.3% of respondents, had plenty of pus cells per HPF.

Table 2 showed that *Escherichia coli* was found in the urine samples of 55.5% of the respondents, and *Enterococcus spp.* was found in the urine samples of 16.6% of the respondents. All other bacterial species were found in less than 10% of the samples.

**Table 2:** Distribution of the bacterial isolates from urine samples among the CKD patients. Data were expressed as frequency and percentage.

Bacteria Isolates		Frequency	Percentage
Gram Negative			
	Proteus spp.	6	1.8%
	Acinetobacter spp.	10	3.1%
	Pseudomonas spp.	12	3.7%
	Enterobacter spp.	24	7.4%
	Klebsiella spp.	26	8.0%
	Escherichia coli	181	55.5%
	Total	259	79.4%
Gram Positive			
	Streptococcus	4	1.2%
	Staphylococcus aureus	9	2.8%
	Enterococcus spp.	54	16.6%
	Total	67	20.6%
Total		326	100.00

Table 3 showed that among all the antibiotics tested, Colistin Sulphate, Polymyxin B, Cefoxitin, Vancomycin, and Linezolid had the highest (100%) sensitivity, followed by Meropenem with 94.94% sensitivity. The Quinolone Group of antibiotics showed the highest resistance: 76.69% for Nalidixic Acid and 62.42% for Ciprofloxacin.

**Table 3:** Distribution of antibiotics according to their sensitivity and resistance to the bacteria isolates causing UTI Data were expressed as frequency and percentage.

Antibiotic		Sensitive		Resistance		Tests Done
		n	%	n	%	N
Penicillin Group						
	Cloxacillin	7	77.78	2	22.22	9
	Ticarcillin	5	62.50	3	37.50	8
	Tazobactam+Piperacillin	16	69.57	7	30.43	23
	Mecillinum	172	72.57	65	27.43	237
	Amoxicillin	82	29.18	199	70.82	281
Quinolone Group						
	Ciprofloxacin	121	37.58	201	62.42	322
	Nalidixic Acid	55	23.31	181	76.69	236
Aminoglycoside Group						
	Netilmicin	206	76.58	63	23.42	269
	Amikacin	171	64.29	95	35.71	266
	Gentamicin	131	40.94	189	59.06	320
Cephalosporin Group						
	Cephradine	5	55.56	4	44.44	9
	Ceftriaxone	130	58.04	94	41.96	224
	Ceftazidime	128	57.14	96	42.86	224
	Cefotaxime	114	51.12	109	48.88	223
	Cefuroxime	114	46.91	129	53.09	243
	Cefoxitin	8	100.00	0	0	8
Meropenem		244	94.94	13	5.06	257
Nitrofurantoin		236	77.63	68	22.37	304
Aztreonam		124	55.61	99	44.39	223
Cotrimoxazole		194	61.78	120	38.22	314
Colistin Sulphate		24	100.00	0	0	24
Polymyxin B		3	100.00	0	0	3
Vancomycin		59	100.00	0	0	59
Linezolid		59	100.00	0	0	59

Table 4 showed that *Acinetobacter* and *Enterobacter* were mostly resistant to aminoglycoside. *E. coli*, *Klebsiella*, *Proteus*, and *Pseudomonas* were mostly resistant to quinolones.

**Table 4:** Antimicrobial resistance pattern of gram-negative bacteria isolates found in urine samples

Antibiotic	Gram Negative Bacteria Isolates in Urine Sample - Frequency (Percentage)					
	Acinetobacter (n = 10)	Enterobacter (n = 24)	E. coli (n = 181)	Klebsiella (n = 26)	Proteus (n = 6)	Pseudomonas (n = 12)
Penicillin	5 (50.00%)	17 (70.83%)	123 (67.96%)	19 (73.08%)	4 (66.67%)	6 (50.00%)
Quinolone	6 (60.00%)	18 (75.00%)	139 (76.80%)	20 (76.92%)	5 (83.33%)	8 (66.67%)
Aminoglycoside	7 (70.00%)	22 (91.67%)	120 (66.30%)	15 (57.69%)	2 (33.33%)	6 (50.00%)
Cephalosporin	6 (60.00%)	7 (29.17%)	79 (43.65%)	12 (46.15%)	4 (66.67%)	5 (41.67%)
Cotrimoxazole	2 (20.00%)	10 (41.67%)	71 (39.23%)	12 (46.15%)	2 (33.33%)	4 (33.33%)
Nitrofurantoin	2 (20.00%)	7 (29.17%)	30 (16.57%)	9 (34.62%)	3 (50.00%)	1 (8.33%)
Aztreonam	0	6 (25.00%)	65 (35.91%)	8 (30.77%)	2 (33.33%)	4 (33.33%)
Meropenem	0	0	5 (2.76%)	0	0	2 (16.67%)

Table 5 shows *Enterococci* and *Staphylococcus aureus* were most resistant to aminoglycoside, 81.48% and 88.89%, respectively. *Streptococcus* was found to be most resistant to cephalosporin (75.00%).

**Table 5:** Antimicrobial resistance pattern of gram-positive bacteria isolates found in urine samples

Antibiotic	Gram-Positive Bacteria Isolates in Urine Sample Frequency (Percentage)		
	*Enterococci* (n = 54)	*Staphylococcus aureus* (n = 9)	*Streptococcus* (n = 4)
Penicillin	31 (57.41%)	7 (77.78%)	2 (50.00%)
Quinolone	43 (79.63%)	6 (66.67%)	2 (50.00%)
Aminoglycoside	44 (81.48%)	8 (88.89%)	1 (25.00%)
Cephalosporin	15 (27.78%)	3 (33.33%)	3 (75.00%)
Cotrimoxazole	13 (24.07%)	5 (55.56%)	1 (25.00%)
Nitrofurantoin	12 (22.22%)	4 (44.44%)	0
Aztreonam	9 (16.67%)	3 (33.33%)	2 (50.00%)
Meropenem	4 (7.41%)	1 (11.11%)	1 (25.00%)

**Figure 2:**
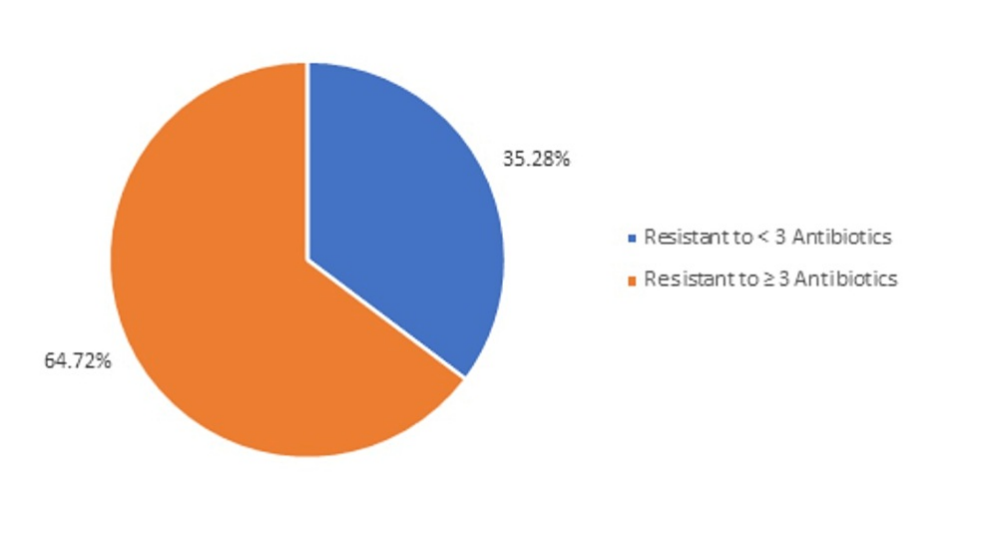
Distribution of bacterial isolates according to multidrug resistance (MDR)

Figure 2 demonstrated that among the 326 urine samples, 115 (35.28%) were found to be resistant to < 3 antibiotics and 211 (64.72%) were found to be resistant to ≥ 3 antibiotics. Thus, 64.72% of the study samples have multi-drug-resistant UTIs.

Table 6 shows that out of the 259 samples with gram-negative isolates, 33.6% were resistant to less than three different groups of antibiotics, and 66.4% were resistant to 3-7 groups of antibiotics. No gram-negative isolate was resistant to all eight groups of antibiotics.

**Table 6:** Distribution of gram-negative bacteria isolates according to their MDR status (N = 259)

Resistances to number of groups of antibiotics	Gram-Negative Bacteria Isolates in Urine Sample Frequency (Percentage)						
	*Acinetobacter* (n = 10)	*Enterobacter* (n = 24)	*E. coli* (n = 181)	*Klebsiella* (n = 26)	*Proteus* (n = 6)	*Pseudomonas* (n = 12)	Total (n = 259)
< 3	3 (30.0%)	7 (29.2%)	62 (34.3%)	9 (34.6%)	2 (33.3%)	4 (33.3%)	87 (33.6%)
3 – 7	7 (70.0%)	17 (70.8%)	119 (65.7%)	17 (65.4%)	4 (66.7%)	8 (66.7%)	172 (66.4%)
> 7	0	0	0	0	0	0	

Table 7 shows that out of the 67 samples with gram-positive isolates, 41.8% were resistant to less than three different groups of antibiotics, 56.7% were resistant to 3-7 groups of antibiotics, and 1.9% were resistant to all eight of the different groups of antibiotics.

**Table 7:** Distribution of gram-positive bacteria isolates according to their MDR status (N = 67)

Resistances to number of groups of antibiotics	Gram-Positive Bacteria Isolates in Urine Sample Frequency (Percentage)			
	*Enterococci* (n = 54)	*Staphylococcus aureus* (n = 9)	*Streptococcus* (n = 4)	Total (n = 67)
< 3	25 (46.3%)	2 (22.2%)	1	28 (41.8%)
3 – 7	28 (51.9%)	7 (77.8%)	3 (75.0%)	38 (56.7%)
> 7	1 (1.9%)	0	0	1 (1.9%)

Table 8 showed that there was a statistically significant (p < 0.05) relationship between the presence of MDR UTI and previous UTI, the history of (H/O) taking an antibiotic(s) within the last six months, and the diabetic status of the respondents.

**Table 8:** Relation of multi-drug-resistant UTI with previous UTI, H/O antibiotic(s) intake within the last six months, and co-morbidities. Chi-square test was done to check for a statistically significant relation. A p-value of < 0.05 was considered statistically significant.

Criteria		MDR Bacteria Isolates	Non-MDR Bacteria Isolates	Significance
History of UTI	Yes	175 (53.7%)	67 (20.6%)	P < 0.05
	No	36 (11.0%)	48 (14.7%)	
History of antibiotic intake	Yes	170 (52.1%)	23 (7.1%)	P < 0.05
	No	41 (12.6%)	92 (28.2%)	
Co-morbidities	CKD with Diabetes Mellitus	123 (37.7%)	44 (13.5%)	P < 0.05
	Non-Diabetic CKD	88 (27.0%)	71 (21.8%)	

## Discussion

This study determined the etiology and factors associated with MDR UTI in patients with CKD and their antibiotic resistance patterns. Among the 326-study population, 130 (39.9%) were male, and 196 (60.1%) were female. Previous studies have shown the prevalence of UTI to be more prevalent among females (50%-60%), which is consistent with our study findings [17].

Past studies have shown that up to 53% of respondents could have had a history of diagnosed UTI in the last six months [18,19], which is closer to current study findings. This history of UTI among the study population is a concerning factor since studies have shown that patients with a history of diagnosed UTI in the past might be at increased risk of contracting UTI in the future [20]. And recurrent UTI causes more inflammatory changes in the renal parenchyma, which is one of the factors in CKD progression.

The majority of respondents had plenty of pus cells per HPF. Gram-negative bacteria isolates were present in 79.4% of the samples, and gram-positive bacteria isolates were present in 20.6% of the samples. Prior studies have shown gram-negative bacteria to be present in 75% of UTI cases [21]. 

Some previous studies represented that among the gram-negative bacteria, the main pathogen responsible for UTI is *Escherichia coli*, followed by other species of *Enterobacteriaceae*, such as *Proteus* and *Klebsiella*, and among the gram-positive pathogens, *Enterococcus* and *Staphylococcus* were the main culprits [22]. The present study results also supported that finding.

Bacteria isolates resistant to ≥ 3 antibiotics were considered multidrug-resistant (MDR), and 64.72% of our study samples were classified as MDR UTI. Previous studies have shown the prevalence of MDR UTI to be 63% [23]. A USA-based study conducted in 2017 showed the presence of multi-drug-resistant bacterial isolates among 69.5% of the study population, which is consistent with our study findings [24]. 

Among the 211 multidrug-resistant (MDR) isolates, 81.5% were gram-negative, and 18.5% were gram-positive. Prior studies have shown gram-negative bacteria isolates to be present in 75% of UTI cases, which is closer to our current study findings [21].

Among the penicillin group of antibiotics, amoxicillin was found to be the drug with the highest resistance, 70.82%, meaning bacteria isolates found in only 29.18% of urine samples were sensitive to amoxicillin. A 2017 study published on PLOS showed amoxicillin to be 78.5% sensitive to bacterial isolates [25]. Another 2012 study shows amoxicillin to be 60%-70% sensitive to various UTI-causing bacteria isolates [26], which is higher than the present study findings. The very high rate of amoxicillin resistance in the Bangladeshi population could be due to the irrational use of antibiotics among the mass population and physicians.

37.58% and 23.31% sensitivity for Ciprofloxacin and Nalidixic Acid, respectively, were revealed in this study. Previous studies have shown ciprofloxacin to have 39.13%-63.63% sensitivity for bacteria isolates and nalidixic acid to have 65.21%-72.72% sensitivity against bacteria isolates in urine, which is higher than the present study findings [26]. Another 2017 study showed Ciprofloxacin to be up to 76.6% sensitive to various isolates, which is higher than our findings [25]. Apart from irrational use, other responsible factors can also be searched for in further study.

From the Aminoglycoside group of antibiotics, Gentamicin showed the lowest (40.94%) sensitivity. A 2012 study showed Gentamicin to be sensitive to 33%-54% of UTI-causing bacteria isolates, which corresponds to the findings of the present study [26]. Another study has shown Gentamicin to be 57.1%-74.8% sensitive against various bacteria isolates, which is higher than the present study findings [25].

In the present study, Nitrofurantoin was found to be 77.63% sensitive, which is slightly lower than previous study findings, where Nitrofurantoin was found to be 85.7%-98.1% sensitive against various bacteria isolates [25,26].

Among all the antibiotics tested, Colistin Sulphate, Polymyxin B, Cefoxitin, Vancomycin, and Linezolid showed the highest (100%) sensitivity, followed by Meropenem, with 94.94% sensitivity. Vancomycin was shown to have 90.2% sensitivity in a 2016 study [27], which is closer to our present study. Previous studies have shown Colistin Sulphate to be 89.5% sensitive to bacteria causing UTI [28]. While none of these studies have found them to be 100% sensitive like the present study, the findings are close enough to support the findings of the present study.

The antimicrobial resistance patterns of gram-negative bacteria isolates (*E. coli*, *Klebsiella*, *Proteus*, and *Pseudomonas*) were most resistant to quinolones (76.80%, 76.92%, 83.33%, and 66.67%, respectively). A 2012 study showed *E. coli* to be resistant to the quinolone group of drugs, up to 72.0% among the Chinese population [29]. Acinetobacter and Enterobacter were found to be most resistant to aminoglycoside at 70% and 91.67%, respectively.

The antimicrobial resistance pattern of gram-positive bacteria isolates was evaluated, and *Enterococci* and *Staphylococcus aureus* were found to be most resistant to Aminoglycoside, 81.48% and 88.89%, respectively.

MDR status was evaluated among gram-negative bacteria. A total of 66.4% of organisms were resistant to 3-7 organisms. In the case of gram-positive organisms, 56.7% were resistant to 3-7 organisms. This is alarming because more than 50% of both gram-positive and gram-negative organisms are resistant to 3-7 antibiotics.

A statistically significant (p < 0.05) relation was found between MDR UTI and previous H/O UTI, H/O taking antibiotics within six months, and the diabetic status of the respondents.

Long-standing diabetes mellitus and poor glycemic control eventually develop immunologic dysfunction, causing defective migration and chemotaxis in polymorphonuclear leukocytes; autonomic neuropathy, resulting in incomplete bladder emptying; and higher glucose concentrations in urine, promoting the growth of pathogenic bacteria. All these factors may contribute to the increased risk of UTI. In a previous study [30], the prevalence of urinary tract infections was significantly higher among diabetic patients (40.2%) than among non-diabetic patients [30]. Frequent prescription of antibiotics, especially broad-spectrum antibiotics, may result in the development of antibiotic-resistant urinary pathogens, specifically multidrug-resistant strains.

Limitations of the study

Due to the purposive sampling technique, there was more chance of bias. The study was done on only one hospital, so the results from this study are only representative of this hospital and not a country-wide finding. Due to the small sample size, all the bacterial species were not properly represented.

## Conclusions

Among the study subjects who were CKD with UTI patients, 64.72% were classified as MDR UTI. *Escherichia coli* was the most prevalent bacterial isolate present in the urine samples, which was 55.5% of all urine samples and 56.4% of all the samples with MDR UTI. History of UTI, history of taking antibiotics within the last six months, and patients with diabetic CKD was found to be significantly (p <0.05) associated with the development of MDR UTI. Penicillin, Quinolone, and Aminoglycoside groups of antibiotics are resistant to most of the bacterial isolates from both gram-negative and gram-positive groups. Colistin Sulphate, Polymyxin B, Cefoxitin, Vancomycin, and Linezolid had the highest sensitivity to bacterial UTI, followed by Meropenem and Nitrofurantoin.

Rational use of antibiotics and correction of risk factors can reduce MDR-UTI among CKD patients. The more patients who suffer from MDR, the greater the expenditure for medication and hospital stays. A future study should be conducted with a larger, population-representative sample size following a simple random sampling technique. This will increase the probability that all the bacterial species will be properly represented in the study and reduce the chance of bias. A multi-center study will also help with getting more country-representative data on the antibiotic resistance pattern of bacterial isolates causing UTIs.
